# Making Sense of Medicine: Bridging the Gap Between Doctor Guidelines and Patient Preferences

**DOI:** 10.5195/jmla.2017.125

**Published:** 2017-04

**Authors:** Margaret Arleen Hoogland

This book’s author works at Johns Hopkins University in the Department of General Internal Medicine. In addition to being a clinician with a focus on internal medicine, Berger has a doctorate in epidemiology, works as a bioethicist, instructs medical students and residents, and sees patients. Berger begins and ends the book by describing what he views as the major components of how medicine is practiced today. He identifies tests, genes, patient involvement, and individualized care as the common ways for treating patients. Berger’s chapters are interesting, are well written, and make sense for people with little medical knowledge or background.

In the middle chapters of the book, Berger describes what he considers to be the five most serious issues in medicine (diabetes, high blood pressure, arthritis, depression, surgery). He does a good job of portraying the role of the health care practitioner, makes suggestions for appropriate times to refer patients to specialists, and provides a general idea of the challenges that patients can present during an appointment. He takes the time to acknowledge the influence of technology and social media on health care but concludes that these influences are often more detrimental than beneficial. Berger advocates for patients to reach decisions themselves by considering the available options and provides minimal input on the decision-making process beyond answering the questions he is asked. His approach could be influenced by mainly treating patients suffering from chronic diseases, who frequently have inadequate health care coverage.

This book outlines prevalent health issues and discusses the problems inherent with acceptable and new ways of providing patient care. The author acknowledges that recent changes in the health insurance system help solve some problems, while creating other problems. Berger provides a realistic view of what internal medicine clinicians, especially those handling chronic diseases, see in clinics, which would be useful for current health care practitioners or medical students considering this specialty.

